# A Prediction Model for Peak Expiratory Flow Derived From Venous Blood Biomarkers and Clinical Factors in Amyotrophic Lateral Sclerosis

**DOI:** 10.3389/fpubh.2022.899027

**Published:** 2022-05-27

**Authors:** Xianghua He, Jiaming Feng, Xue Cong, Hongyan Huang, Quanzhen Zhao, Qiuyan Shen, Fang Xu, Yanming Xu

**Affiliations:** ^1^Department of Neurology, West China Hospital, Sichuan University, Chengdu, China; ^2^Department of Neurology, Jiangbin Hospital, Nanning, China; ^3^West China Clinical Medical College, Sichuan University, Chengdu, China; ^4^West China School of Public Health, Sichuan University, Chengdu, China

**Keywords:** Amyotrophic lateral sclerosis, peak expiratory flow, prediction model, respiratory function, venous blood parameters

## Abstract

Although peripheral venous blood biomarkers are related to respiratory function in Amyotrophic lateral sclerosis (ALS) patients, there are still few prediction models that predict pulmonary function. This study aimed to investigate the venous blood biomarkers associated with respiratory function in patients with ALS from southwest China and to create prediction models based on those clinical biomarkers using logistic regression. A total of 319 patients with ALS from the retrospective cohort and 97 patients with ALS from the prospective cohort were enrolled in this study. A multivariable prediction model for the correlation between peak expiratory flow (PEF) and hematologic, biochemical laboratory parameters, and clinical factors in patients with ALS was created. Along with female patients, bulbar-onset, lower body mass index (BMI), later age of onset, lower level of creatinine, uric acid, triglyceride, and a higher level of high-density lipoprotein cholesterol (HDL_C) were related to reduced PEF. The area under the receiver operating characteristics (ROC) curve is.802 for the test set and.775 for the validation set. The study constructed a multivariable prediction model for PEF in patients with ALS. The results can be helpful for clinical practice to predict respiratory impairment.

## Introduction

Amyotrophic lateral sclerosis (ALS) is a rapidly progressive neurodegenerative disease clinically characterized by loss of bulbar and limb function (1). Recent studies have shown that the average onset age of ALS is 51–66 years (2). Men have a higher risk of developing ALS than women. Studies reported a male to female ratio of 1:2 (2). Patients with ALS usually die from respiratory failure 3–5 years after the symptom onset (3).

To monitor the health status of patients with ALS, close clinical and functional follow-up with particular care for respiratory monitoring was recommended for patients with ALS (4).

Peak expiratory flow (PEF) can assess the strength of respiratory muscles and can evaluate the efficacy of clearing the bronchial tree (5). The study reported that 85% of patients with ALS would develop dysphagia and impaired secretion management, leading to impairment of mucociliary clearing and airway protection (6), which resulted in malnutrition, dehydration, and aspiration pneumonia (7). PEF can be used as a tool for assessing cough ability for patients who underwent lung surgery (8), pulmonary function assessment before lung surgery (9, 10), and airway clearance in neurodegenerative disease (11). Recent studies reported that respiratory impairments in patients with ALS demonstrated reduced peak expiratory flow (PEF) rates compared to healthy controls (12, 13), implying that PEF may be a good screen tool for identifying patients with ALS at risk for aspiration (13). Moreover, the authors found that PEF was an independent factor for survival (14) and positively correlated with the revised functional rating scale score (ALSFRS-R), indicating a predictive value for survival and disease severity in patients with ALS (15).

For patients in the final stages of ALS, the pulmonary function tests were hardly carried out due to their inability to perform the tests. Thus, it is necessary to explore more easily accessible and low-cost parameters for the prediction of respiratory function. Venous blood parameters have been related to respiratory impairment and disease severity in patients with ALS in several studies (16–20). To the best of our knowledge, no studies of venous blood biomarkers associated with PEF in patients with ALS were seen.

We aimed to develop and validate a predictive model based on a new panel of eleven variables analyzed in venous blood parameters of patients with ALS, along with their clinical characteristics. The multivariable logistic regression model created in this study can predict the impairment of PEF in patients with ALS.

## Materials and Methods

### Patients

This is a retrospective and prospective study. Patients from a large, regional, and referral ALS clinic in Southwest China, were enrolled in the study. In the retrospective cohort, 319 patients with ALS were registered from 1 January 2015 to 30 December 2020. In the prospective cohort, 97 participants from 1 March 2017 to 30 October 2021 were enrolled. All the participants fulfilled the revised El Escorial criteria for probable or definite ALS (21, 22). We excluded patients with other medical or neurological diseases and patients with missing baseline hematologic and biochemical values. Patients with manifestations of neoplastic disorders were also excluded. This study was approved by the Ethical Committee of West China Hospital of Sichuan University.

### Clinical Variables

In this study, the relevant demographic and clinical data were collected, including age, age at ALS onset and diagnosis, sex, onset body region, and blood pressure. BMI was calculated at the time of pulmonary function tests. High blood pressure was defined as systolic blood pressure (SBP) of ≥140 mmHg and/or diastolic blood pressure (DBP) of ≥90 mmHg in this study.

Hematologic and biochemical laboratory parameters were obtained using an automated hematology analyzer (KX-21 N, Sysmex America, Lincolnshire, IL, USA), and data on platelet count (PLT), neutrophil count (NEUT#), lymphocyte count (LYMPH#), monocyte count (MONO#), eosinophil count (EO#), basophil count (BASO#), albumin (ALB), glucose (GLU), triglyceride (TG), urea (UREA), cholesterol (CHOL), uric acid (URIC), high-density lipoprotein cholesterol (HDL_C), creatinine (CREA), and creatine kinase (CK) were obtained. PLR was defined as the ratio of PLT to LYMPH#. Using electronic spirometry, PEF was measured with the patient in a standing position.

### Statistical Analysis

#### Correlation Analysis

Continuous variables with normal distribution were reported as mean (standard deviation [SD]); non-normal variables were presented as median (interquartile range (IQR), 25 to 75%). The linear correlations of these variables and the respiratory function index (PEF) were calculated by Spearman. For categorical variables, the correlations were performed by the nonparametric test (Wilcoxon rank-sum test). For normal distribution data, differences between the groups who had a PEF of <5.50 or not were performed by the Independent Samples *t-*test. For non-normal distribution, differences were estimated using the Mann-Whitney test or chi-squared tests.

#### The Training Set and the Test Set

**Figure 1:**
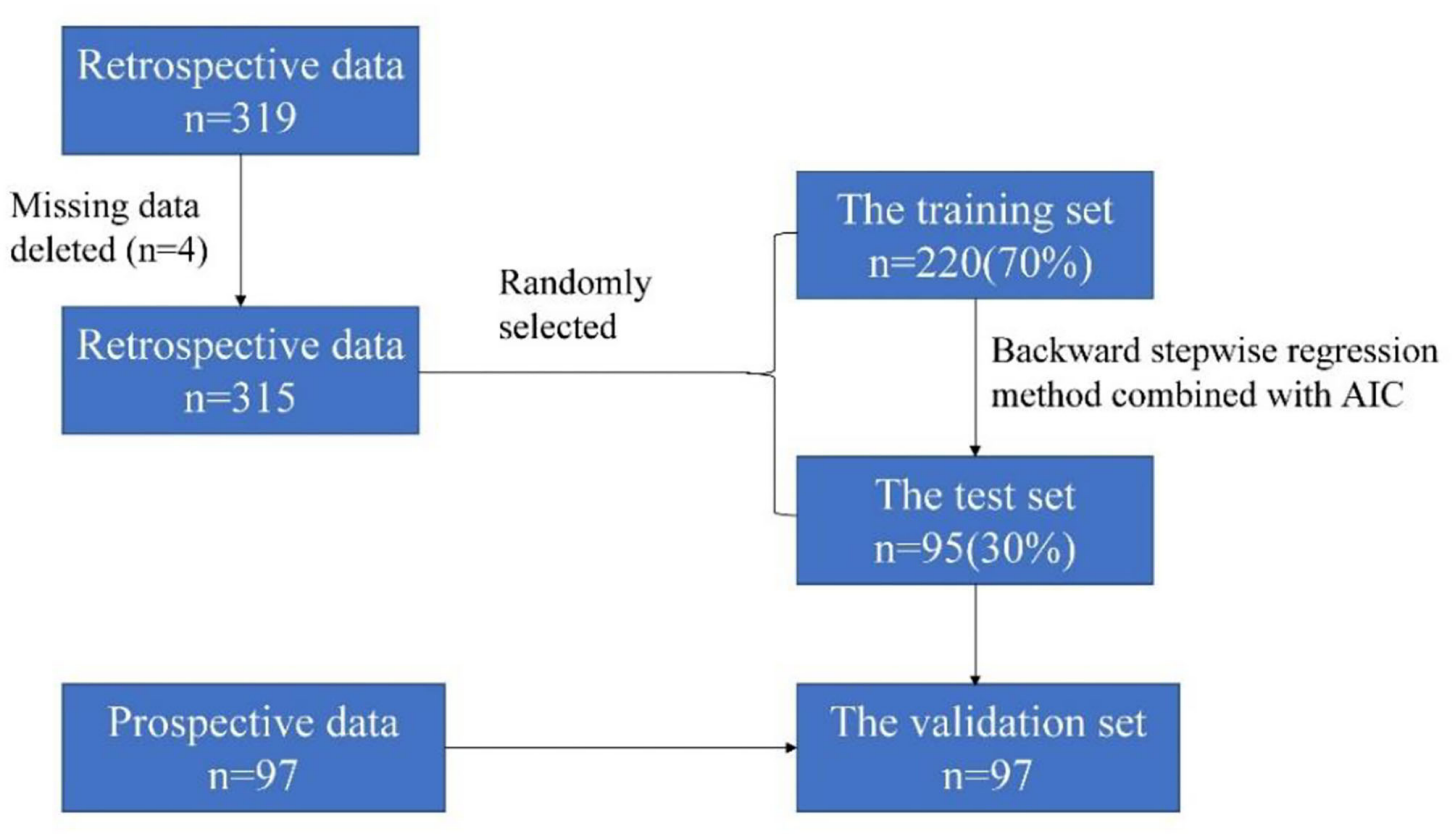
The flowchart in this study.

The retrospective cohort was divided into two independent sets: the training set and the test set. The training set included 70% (220/319) of the retrospective cohort, randomly selected from the cohort. The test set included 30% (95/319) of the retrospective cohort. Models were developed based on the characteristics of the training set. The test set was used to validate the models (Figure 1). Partition of the data set was realized by the sample() function in R. In the partitioning, the set.seed() function was used to generate a sequence of random numbers.

#### Logistic Regression

A total of 23 variables, namely, gender, onset age, site of onset, diagnostic level, height, BMI, Blood pressure, PLT, PLR, NEUT#, LYMPH#, MONO#, EO#, BASO#, ALB, GLU, UREA, CREA, URIC, TG, CHOL, HDL_C, and CK, were included to construct the model. Based on the results of the backward stepwise regression method, combined the with Akaike information criterion (AIC), the heatmap of PEF for predicting peak expiratory flow impairment in patients with ALS was established. Receiver operating characteristic (ROC) curves and calibration curves were drawn to evaluate the predictive accuracy. Then, we tested the model in the validation set. All statistical analyses were performed using SPSS 24.0 (IBM, Chicago, IL, USA) and R 4.1.0 (www.rproject.org).

## Results

Of the 416 patients with Amyotrophic lateral sclerosis included in the study, there were 57 males (51.35%) in the retrospective cohort and 16 males (47.06%) in the prospective cohort with PEF of <5.5 L/s, while 54 females (48.65%) in the retrospective cohort and 18 females (52.94%) in the prospective cohort with PEF of <5.5L/s (Table 1). Similarly, there were significant age differences in onset age, BMI, level of PLR, CREA, TG, and HDL_C (all *P* < 0.05) when stratified by PEF either in the retrospective cohort or in the prospective cohort (Table 1).

**Table 1:** Clinicodemographic data of Amyotrophic lateral sclerosis patients stratified by whether their PEF is ≥5.50 L/s.

	**The retrospective cohort**			**The prospective cohort**		
**Variable**	**PEF ≥5.5 (*n* = 208)**	**PEF <5.5 (*n* = 111)**	***P***	**PEF ≥5.5 (*n* = 63)**	**PEF <5.5 (*n* = 34)**	***P***
**Gender**						
Male	173 (83.17%)	57 (51.35%)	<0.001	43 (68.25%)	16 (47.06%)	0.041
female	35 (16.83%)	54 (48.65%)		20 (31.75%)	18 (52.94%)	
age	52.59 ± 10.39	59.64 ± 10.40	<0.001	53.37 ± 10.57	61.4 4± 11.83	0.001
onset age	51.44 ± 10.38	58.26 ± 10.91	<0.001	52.35 ± 10.39	60.47 ± 11.90	0.001
**Hypertension**						
SBP	131.18 ± 16.88	133.59 ± 16.74	0.229	130.03 ± 21.43	129.18 ± 17.80	0.843
DBP	88.31 ± 13.24	85.79 ± 11.96	0.098	85.30 ± 13.04	84.65 ± 13.25	0.655
weight	60.00 (13.00)	55.00 (12.00)	<0.001	60.92 ± 8.97	52.88 ± 7.35	<0.001
height	1.63 ± 0.075	1.57 ± 0.074	<0.001	1.62 ± 0.07	1.58 ± 0.08	0.012
BMI	22.93 ± 2.98	22.16 ± 3.07	0.03	23.21 ± 2.70	21.28 ± 2.96	0.002
**Site of onset**						
limb onset	179 (86.06%)	80 (72.73%)	0.007	49 (77.78%)	26 (76.47%)	0.883
bulbar onset	23 (11.06%)	27 (24.54%)		14 (22.22%)	8 (23.53%)	
other	6 (2.88%)	3 (2.73%)		0	0	
**Diagnostic level**						
probable	102 (49.04%)	53 (47.75%)	0.826	46 (73.02%)	24 (70.59%)	0.799
definite	106 (50.96%)	58 (52.25%)		17 (26.98%)	10 (29.41%)	
**PLT**	177.50 (79.00)	195.00 (78.00)	0.052	167.00 (80.00)	188.50 (54.50)	0.084
**NEUT#**	3.39 (1.51)	3.41 (1.67)	0.644	3.21 ±1.06	3.44 ± 1.03	0.552
**LYMPH#**	1.79 (0.62)	1.62 (0.79)	0.035	1.60 (0.79)	1.39 (0.87)	0.165
**PLR**	101.26 (52.94)	114.52 (62.55)	0.002	94.04 (61.65)	128.60 (66.52)	0.027
**MONO#**	0.41 (0.16)	0.38 (0.19)	0.283	0.41 ± 0.12	0.38 ± 0.14	0.632
**EO#**	0.12 (0.12)	0.12 (0.12)	0.508	0.14 (0.16)	0.14 (0.15)	0.797
**BASO#**	0.03 (0.02)	0.03 (0.02)	0.293	0.02 (0.01)	0.02 (0.01)	0.720
**ALB**	42.80 (4.30)	41.70 (4.60)	0.005	41.80 (3.60)	42.40 (6.15)	0.731
**GLU**	4.75 (0.83)	4.87 (0.71)	0.295	4.72 (0.77)	4.71 (0.60)	0.518
**UREA**	5.00 (1.50)	5.30 (2.01)	0.65	5.50 (2.30)	5.60 (2.18)	0.655
**CREA**	62.00 (16.50)	53.00 (17.00)	<0.001	62.00 (17.00)	52.50 (32.00)	0.049
**URIC**	316.50 (84.00)	295.00 (104.00)	0.003	302.00 (100)	266.50 (92.75)	0.087
**TG**	1.33 (1.03)	1.13 (0.71)	0.007	1.29 (0.93)	1.08 (0.48)	0.004
**CHOL**	4.64 ± 0.85	4.61 ± 0.91	0.814	4.34 ± 0.73	4.88 ± 1.18	0.001
**HDL_C**	1.16 (0.42)	1.37 (0.46)	<0.001	1.19 (0.31)	1.37 (0.60)	<0.001
**CK**	180.00 (217.00)	145.00 (127.00)	0.002	142.00 (157.0)	111.50 (97.5)	0.074

*SBP, systolic blood pressure; DBP, diastolic blood pressure; BMI, body mass index; PLT, platelet count; NEUT#, neutrophil count; LYMPH#, lymphocyte count; PLR, the ratio of PLT to LYMPH#; MONO#, monocyte count; BASO#, basophil count; ALB, albumin; GLU, glucose; UREA, urea; CREA, creatinine; URIC, uric acid; TG, triglyceride; CHOL, cholesterol; HDL_C, high-density lipoprotein cholesterol*.

**Figure 2:**
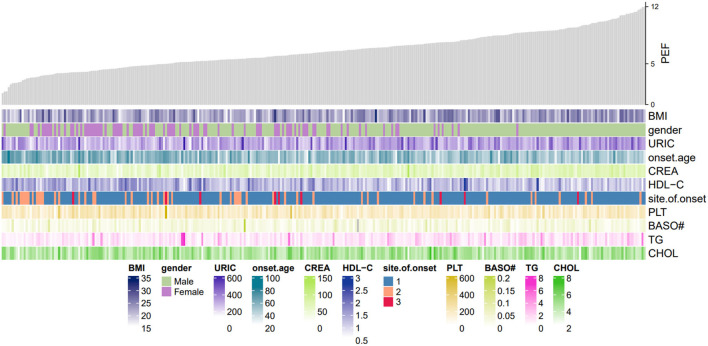
Heatmap of PEF in this study. The gray shistogram represents values of PEF. Below the histogram are factors in the logistic regression model equation. The brightness of the color varied upon the value for continuous variables. PDF, peak expiratory flow; URIC, uric acid; BMI, body mass index; CREA, creatinine; HDL_C, high-density lipoprotein cholesterol; BASO#, basophil count; PLT, platelet count; TG, triglyceride; CHOL, cholesterol.

We developed a multivariable logistic regression model for the association between the hematologic, biochemical laboratory parameters, clinical factors, and PEF in patients with ALS in the test set. The regression equation was developed from the estimate values obtained by z-value and is reported in Table 2. Moreover, we constructed a heatmap to show the relation between these variables in the equation and PEF (Figure 2).

**Table 2:** Variable in the prediction model by backward stepwise regression method combined with Akaike information criterion (AIC) for logistic regression equation.

**variable**	**Estimate**	**Std.error**	**z-value**	**Degree of freedom**	***P*-value**
(Intercept)	−3.483	2.386	−1.46	1	0.144
Gender (female)	1.601	0.450	3.558	1	3.73E−04
Site.of.onset 2	1.373	0.505	2.718	1	0.007
Site.of.onset 3	0.695	1.239	0.561	1	0.575
BMI	−0.106	0.071	−1.499	1	0.134
PLT	0.005	0.003	1.857	1	0.063
‘BASO#‘	−14.968	10.708	−1.398	1	0.162
CREA	−0.041	0.014	−2.842	1	0.004
URIC	0.004	0.003	1.717	1	0.086
TG	0.366	0.234	1.562	1	0.118
CHOL	−0.483	0.236	−2.045	1	0.041
‘HDL-C‘	1.363	0.719	1.897	1	0.058
Onset.age	0.088	0.019	4.723	1	2.33E−06

*Site.of.onset 2, bulbar onset; site.of.onset 3, the onset site except for limb onset and bulbar onset; BMI, body mass index; PLT, platelet count; BASO#, basophil count; CREA, creatinine; TG, triglyceride; URIC, uric acid; CHOL, cholesterol; HDL_C, high-density lipoprotein cholesterol*.

Model PEF = −3.483+1.601 Gender (Female) + 1.373 Site.of.onset 2 (bulbar onset) or.695 Site.of.onset 3 (except for limb onset and bulbar onset) −0.106 BMI +0.005 PLT −14.968 ‘BASO#' −0.041 CREA level +0.004 URIC level +0.366 TG level −0.483 CHOL level + 1.363 HDL_C level +0.088 Onset.age (Table 2).

**Figure 3:**
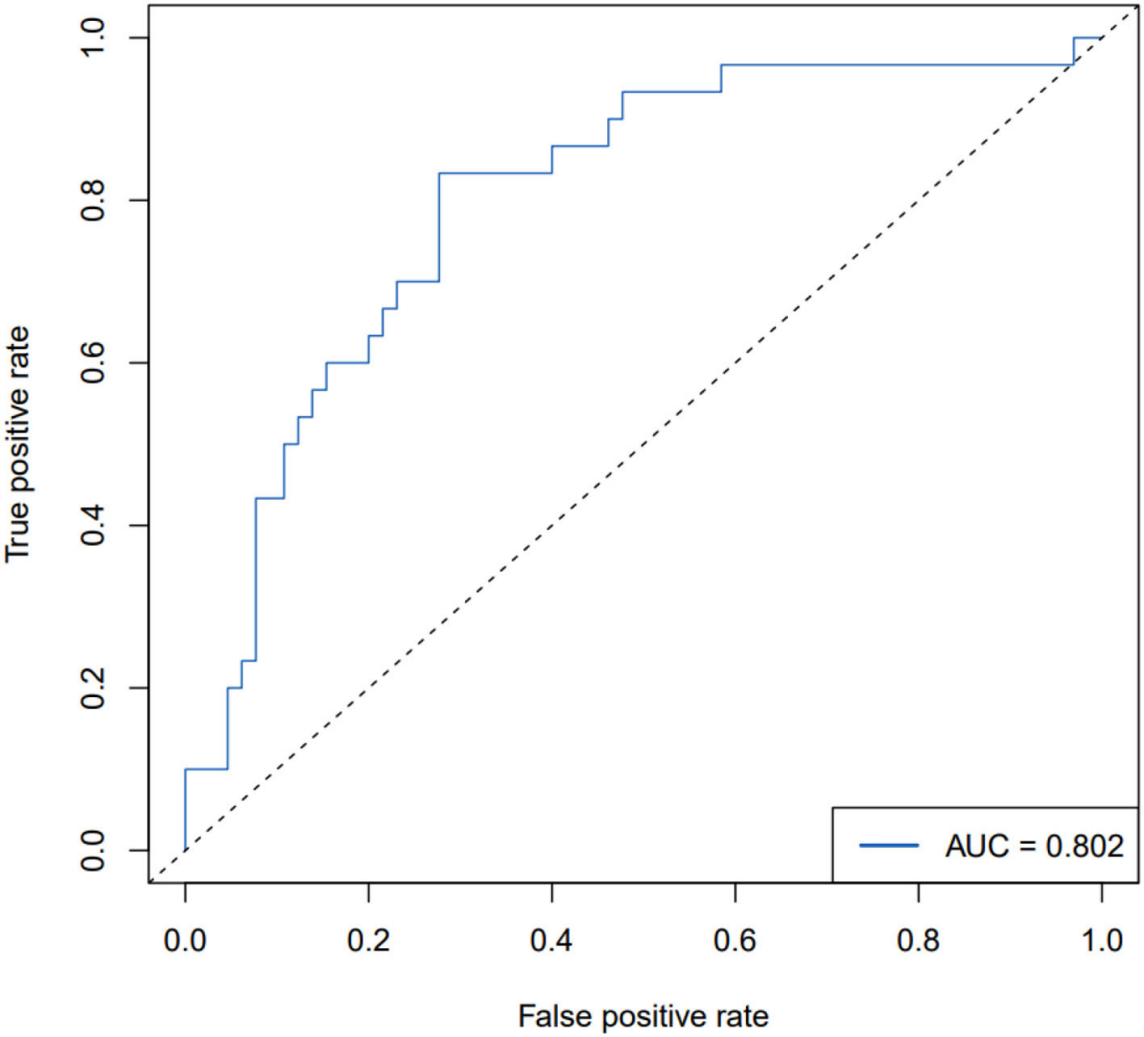
ROC curve for the backward stepwise regression method combined with the AIC model for predicting PEF impairment in patients with ALS in the test set.

**Figure 4:**
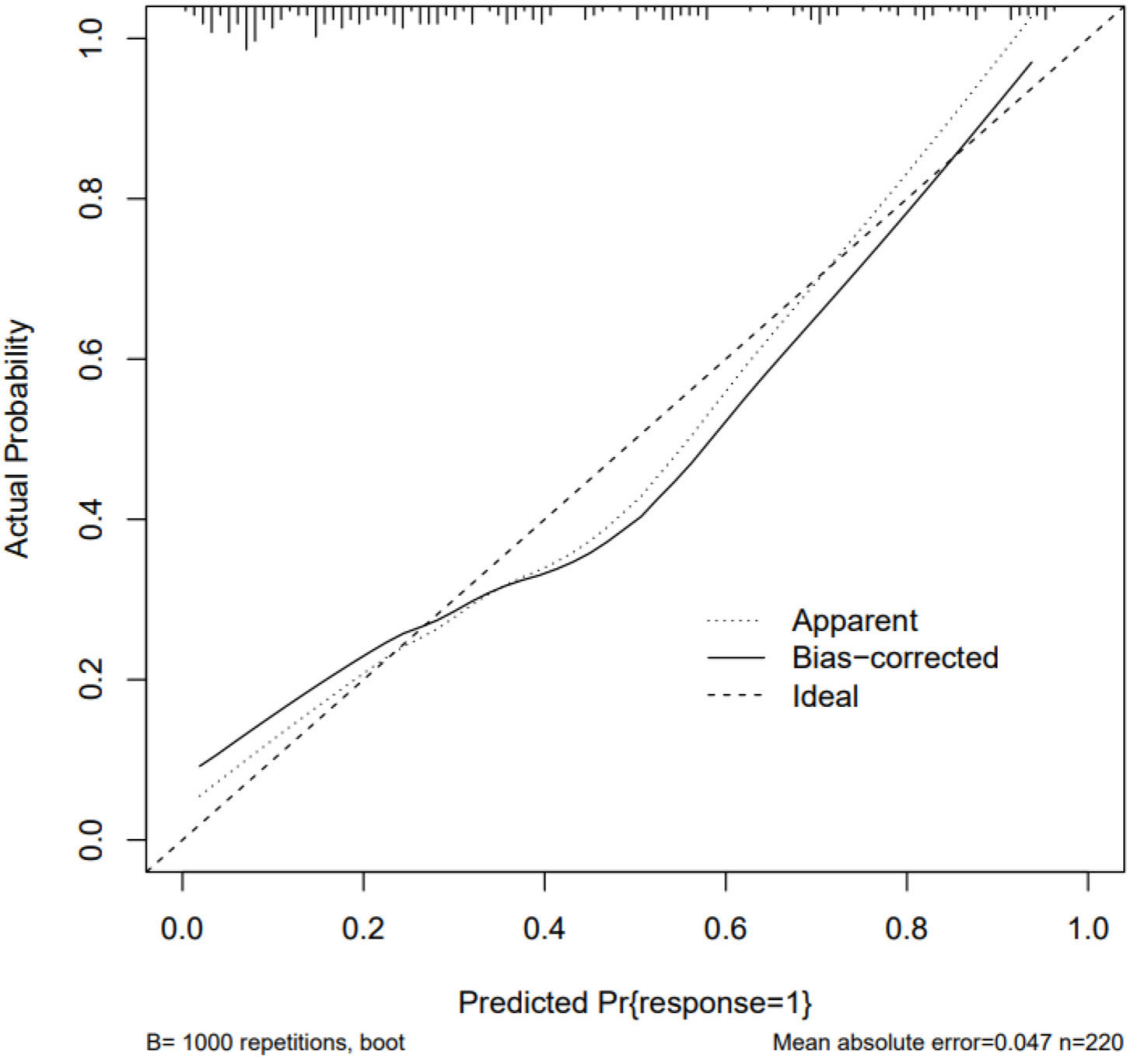
The calibration curve for the model in the training set.

**Figure 5:**
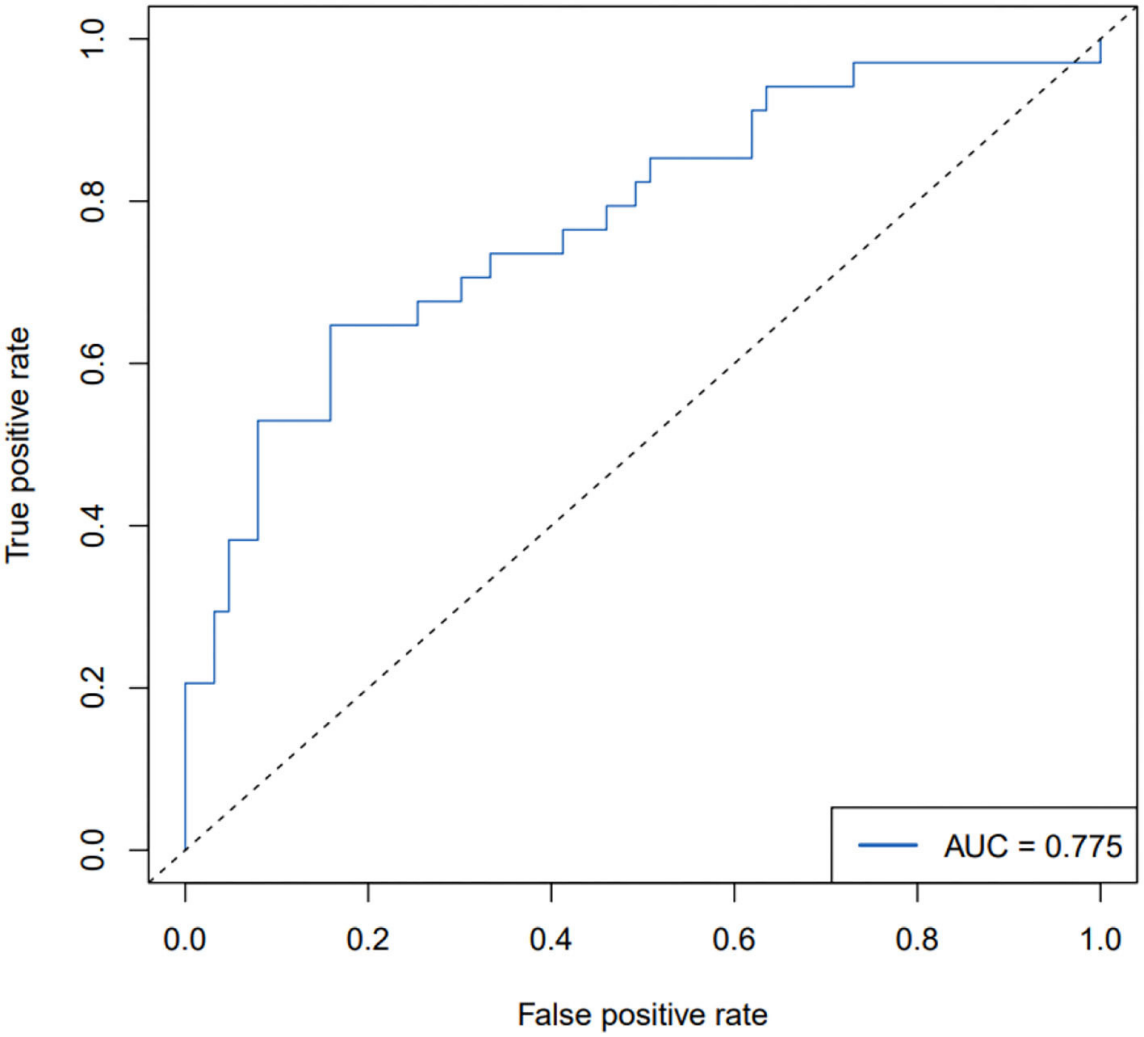
Receiver operating characteristic (ROC) curve of PEF in the validation set.

The ROC curve, with 11 predictive factors, revealed that the area under the curve was 80.2% in the test set (Table 3; Figure 3). And the ROC curve has a standard error of.049 with a 95% confidence interval (CI) of.707–0.898 in the test set. Furthermore, the calibration curve was also constructed (Figure 4). The calibration curves further indicated good calibration power. To validate the model, we tried to predict the data from the prospective cohort and calculated the ROC curve, which yielded a concordance statistic of.775 (95% CI,0.674–0.877) (Figure 5).

**Table 3:** ROC curve for the test set and the validation set.

	**The test set**	**The validation set**
The area under the ROC curve (AUC)	0.802	0.775
Standard error	0.049	0.052
95% Confidence interval	0.707–0.898	0.674–0.877
Z-statistic	6.197	5.326
Significance level P (Area=0.5)	5.77E−10	1.01E−07

## Discussion

Since most patients with ALS in their advanced phase would develop respiratory impairment, it is important for clinicians to evaluate the respiratory function status. To date, there are few studies about models predicting respiratory function in patients with ALS (16, 23). Our study created and validated a multivariable logistic model for the association between PEF and the venous blood biomarkers and clinical factors in patients with ALS. Our single-site study revealed that PEF is related to ALS characteristics, as well as hematologic and biochemical laboratory parameters, and further verification is needed in external data sets with a large sample. To the best of our knowledge, this is the first study of a prediction model for PEF in ALS.

The PEF decreases in neuromuscular diseases (24) and showed excellent discriminant ability to evaluate the presence of aspiration pneumonia (13). Furthermore, the peak cough expiratory significantly correlates with bulbar impairment in patients with ALS (25) and maybe a good indicator to discriminate between ALS with “non-invasive ventilation (NIV) indication” and those with “no NIV indication yet” (26). The prediction model in our study consists of variables that are easy to get. Therefore, it can be a preliminary screening tool for the severity of the disease quickly and be used to evaluate the cough ability of patients with ALS in the outpatient department. The PEF prediction model may be a good screen tool for clinical utility.

In our study, patients with ALS with a lower level of TG, and a higher level of HDL_C showed a trend of reduced PEF. Also, in the prospective cohort, PEF was inversely associated with cholesterol. This study has not confirmed previous research, which found that lower serum lipid levels are related to reduced functional vital capacity (16). Another study from Japan reported that forced vital capacity (FVC) was inversely correlated with serum total cholesterol (17). Prospective cohort studies with a larger sample size are warranted to clarify the relationship between serum lipid and respiratory function.

In contradiction to previous research (23), we found that lower levels of creatinine and uric acid were associated with decreased PEF, but not independent predictors of PEF. Several studies have shown that low levels of serum creatinine and uric acid have been associated with poor survival, and faster functional decline (18–20, 27). One possibility is that patients with ALS with short survival have a faster respiratory decline. These patients have decreased levels of creatinine and uric acid at the same time, ultimately resulting in a low level of creatinine and uric acid-related to impaired respiratory.

Dysphagia in patients with ALS had a close relationship with respiratory impairment (13). A prospective study demonstrated that not only bulbar-onset but also most limb-onset patients with ALS had problems with dysphagia (6). Moreover, about 20% of spinal-onset patients with ALS, who did not show dysphagia at diagnosis, had initial respiratory impairment (28), highlighting the importance of evaluating PEF at the beginning of enrollment. In this study, female patients with ALS with bulbar-onset, later age at onset, and lower BMI tended to have reduced PEF in our study. Age at onset, onset region, and weight loss were factors independently influencing survival in patients with ALS (28–30). Further studies are needed to investigate the relationship between bulbar-onset, age at onset, BMI, and PEF.

Our results should be interpreted with caution due to several limitations. Firstly, our retrospective cohort could not analyze biomarkers potentially significant to the risk of PEF. For example, lipid and uric acid values collected at admission might be affected by the other conditions that coexisted with ALS, which we did not analyze. Secondly, the validation data set in our study was not technically an external data set, thus, external validation is needed. Thirdly, the prediction model is only suitable for probable and definite patients with ALS. Additionally, since the model is derived from the retrospective cohort and a single medical center, it should be tested and verified on larger prospective cohorts to evaluate the validity and predictability.

In conclusion, our study constructed a multivariable logistic regression equation of PEF derived from venous blood biomarkers and clinical factors in patients with ALS. The finding needs to be verified in prospective and multi-site studies.

## Data Availability Statement

The original contributions presented in the study are included in the article/supplementary material, further inquiries can be directed to the corresponding author/s.

## Ethics Statement

The studies involving human participants were reviewed and approved by Sichuan University. The patients/participants provided their written informed consent to participate in this study. Written informed consent was obtained from the individual(s) for the publication of any potentially identifiable images or data included in this article.

## Author Contributions

XH: conceptualization, data curation, formal analysis, methodology, software, validation, visualization, writing-original draft, and writing-review and editing. JF, XC, QZ, QS, and FX: data curation, formal analysis, and methodology. HH: data curation, formal analysis, methodology, and funding acquisition. YX: conceptualization, funding acquisition, project administration, supervision, and writing-review and editing. All authors contributed to the article and approved the submitted version.

## Funding

This work was supported by the Basic Conditions Platform Construction Project of the Sichuan Science and Technology Department (2019JDPT0015), the 1.3.5 Project for disciplines of excellence, West China Hospital, Sichuan University (ZYJC18003), and the Miaozi Project in Science and Technology Innovation Program of Sichuan Province (2020JDRC0057).

## Conflict of Interest

The authors declare that the research was conducted in the absence of any commercial or financial relationships that could be construed as a potential conflict of interest.

## Publisher's Note

All claims expressed in this article are solely those of the authors and do not necessarily represent those of their affiliated organizations, or those of the publisher, the editors and the reviewers. Any product that may be evaluated in this article, or claim that may be made by its manufacturer, is not guaranteed or endorsed by the publisher.
